# Green solvents and technologies for oil extraction from oilseeds

**DOI:** 10.1186/s13065-017-0238-8

**Published:** 2017-01-23

**Authors:** S. P. Jeevan Kumar, S. Rajendra Prasad, Rintu Banerjee, Dinesh K. Agarwal, Kalyani S. Kulkarni, K. V. Ramesh

**Affiliations:** 1 ICAR-Indian Institute of Seed Science, Maunath Bhanjan, Uttar Pradesh 721302 India; 2 Microbial Biotechnology and Downstream Processing Laboratory, Indian Institute of Technology, Kharagpur, West Bengal 721302 India; 3 ICAR-Indian Institute of Rice Research, Rajendra Nagar, Hyderabad, 500030 India

**Keywords:** Aqueous enzyme assisted extraction (AEAE), Green solvents, Ionic liquids, Terpenes

## Abstract

Oilseeds are crucial for the nutritional security of the global population. The conventional technology used for oil extraction from oilseeds is by solvent extraction. In solvent extraction, *n*-hexane is used as a solvent for its attributes such as simple recovery, non-polar nature, low latent heat of vaporization (330 kJ/kg) and high selectivity to solvents. However, usage of hexane as a solvent has lead to several repercussions such as air pollution, toxicity and harmfulness that prompted to look for alternative options. To circumvent the problem, green solvents could be a promising approach to replace solvent extraction. In this review, green solvents and technology like aqueous assisted enzyme extraction are better solution for oil extraction from oilseeds. Enzyme mediated extraction is eco-friendly, can obtain higher yields, cost-effective and aids in obtaining co-products without any damage. Enzyme technology has great potential for oil extraction in oilseed industry. Similarly, green solvents such as terpenes and ionic liquids have tremendous solvent properties that enable to extract the oil in eco-friendly manner. These green solvents and technologies are considered green owing to the attributes of energy reduction, eco-friendliness, non-toxicity and non-harmfulness. Hence, the review is mainly focussed on the prospects and challenges of green solvents and technology as the best option to replace the conventional methods without compromising the quality of the extracted products.

## Background

Conventional oil extraction from oilseeds has been performed by hydraulic pressing, expeller pressing and solvent extraction (SE) [1]. Among these methods, solvent extraction has been widely adapted for economical and practical concerns. Before performing solvent extraction the oilseeds are processed (flaked, cracked, ground or pressed) to suit for the enhanced oil recovery by solvent extraction. In SE process, the oilseeds are washed with hexane, thereafter the hexane is separated from oil by evaporation and distillation [2]. Hexane has been widely used for oil extraction because of easy oil recovery, narrow boiling point (63–69 °C) and excellent solubilizing ability [3].

In contrary, while in extraction and recovery processes, hexane is released into the environment that react with the pollutants to form ozone and photo chemicals [4]. Moreover, several studies revealed that hexane affects neural system when inhaled by humans because of solubility in neural lipids. Toxicity has been observed in piglets fed with de-fatted meal containing residual hexane which was left over after the process [5]. Therefore, health perspective, safety and environment concerns have triggered to look for a substitute to *n*-hexane without compromising the yield of oil. Hence, green solvents coupled with technology are a viable alternative for oil extraction.

Green solvents and technology are aimed to develop an environment friendly process with simultaneous reduction of pollutants [6, 7] for oil extraction. Hence, green technology such as aqueous enzymatic extraction (AEE) coupled with green solvents have huge potential to replace *n*-hexane without any compromise in oil recovery from the process. In addition, the opportunities and challenges of AEE have been given comprehensively to understand the merits and de-merits of the technology.

## Oil extractions by green solvents (GS)

**Fig. 1:**
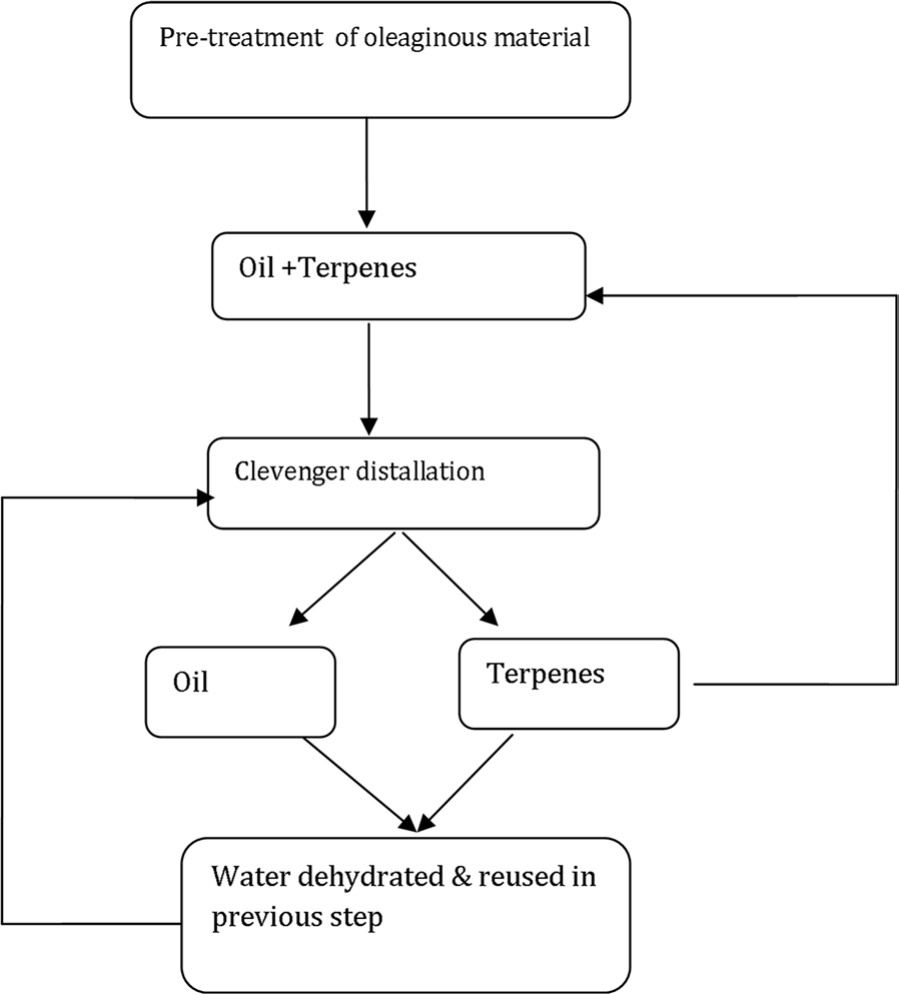
Schematic diagram of oil extraction from oilseeds using terpenes as solvent. (Adapted from [1, 8, 54])

Green solvents are derived either from naturally (water and CO_2_) or agricultural residues (terpenes) or petroleum sources, which have good solubilizing properties like conventional solvents. Recent advances on ‘green’ approaches have great impetus in oil industry because of green solvents i.e., terpenes (d-limonene, *p*-cymene and α-pinene). Terpenes are isoprene units (C_5_H_8_) derived chiefly from agriculture sources. For example, d-limonene is derived from citrus peels and employed in many applications. Similarly, *p*-cymene and α-pinene are derived from tree oils and pine forests respectively. Interestingly, these solvents have good Hansen solubility properties (HSP) to dissolve the like molecules. To determine the behavior of given solvent, Hansen has proposed three properties which is also called Hansen properties based on the energy of dispersive (δ_d_), dipolar (δ_d_) and hydrogen bond forces (δ_h_), between the molecules [8]. In a study, the terpenes were found to possess the characteristics of *n*-hexane that substantiate the capability to dissolve the like molecules (Fig. 1). Moreover, terpenes are not only safer due to higher flash point, but also have slightly higher dissociating power due to slight differences in the dielectric constant in comparison with *n*-hexane [9].

## Ionic liquids

Ionic liquids are non-aqueous salt solution that comprise both anions and cations which can be maintained in a liquid state at moderate temperatures (0–140 °C) [10, 11]. Ionic liquids are considered as green solvents or green ‘designer’ solvents for their manifold applications in petroleum and oil industry. Ionic liquids are eco-friendly in nature as these do not have the detectable vapor pressure, as a result, no pollution. In addition, these are non-flammable, and remain in liquid state for wide range of temperatures [12]. As these solvents possess both the ions and versatile physico-chemical characteristics, these have allowed to design a suitable solvent with specific conductivity, hydrophobicity, polarity, and solubility based on the nature of solute for efficient recovery [13]. Interestingly, because of these properties about 600 molecular solvents were employed in various processes [14].

Ionic liquids were used as solvent for extraction, catalysis and synthesis of various compounds. These can also be used as a co-solvent for enzyme, medium for several reactions, biphasic system separations etc., [15]. However, studies on application of ionic liquids for oil extraction are scanty and needs to substantiate the technical and economical viability. Ma et al. [16] studied the extraction of essential oils using ionic liquids from *Schisandra chinensis* Baill fruit and projected that the ionic liquid coupled with microwave have reduced time, energy and eco-friendly [16]. In other study, the ionic liquid was used as a co-solvent for bio-oil extraction in a single step from microalgae [17]. However, a meta-analysis study reported that the IL’s should be chosen carefully and need to understand their adverse effects [18]. Although, this method is promising but it needs more studies to substantiate the hypothesis of oil extraction from ionic liquids. Another promising green solvent such as switchable solvent has showed potential for oil extraction from soy bean flakes [19]. In addition, super critical fluid, deep eutectic solvents, natural deep eutectic solvents and supramolecular solvents are gaining wide interest and there is a need to study their applicability in oil extraction [11, 20].

## Green techniques for oil extraction from oilseeds

### Aqueous enzymatic extraction (AEE)

Aqueous extraction involves water as a medium to extract the oil from oilseeds. It is well known that the lipid molecules are amphipathic in nature and the water soluble components diffuse into water which culminates into emulsion formation [21]. The emulsified oil in water can be de-emulsified by changing the temperature or deploying enzymes. Hence, in the process of AEE, enzymes are involved which segregate the desired extracted constituent without any damage. Recent investigations have unraveled the tremendous potential of AEE [22]. Moreover, this process is environmental-friendly, safer, healthier, simultaneous oil and protein extraction can be done without compromising the quality. In addition, it is cost-effective as consumption of solvent is reduced and is effective in removal of anti-nutritional factors, toxins and avoid degumming process [23–25]. These several merits make AEE a promising green technique not only for oilseed processing but also to extract the desired compound. The differences between solvent extraction (SE) and enzyme assisted extraction are given in Table 1.

**Table 1:** Comparison of solvent extraction (SE) and aqueous assisted enzymatic (AAE) methods

Parameter	Solvent extraction	Aqueous assisted enzymatic
Nature of the process	Non-environment friendly	Environment friendly
	Toxic	Non-toxic
Solvents used	*n*-Hexane	Green solvents
Energy efficiency	Energy demanding process due to consumption of oil	Less energy demand process
Co-product quality	Poor quality due to operational conditions at higher temperature and pressure	Food quality grade due to mild operational conditions
Degumming	It is essential because of phospholipids	Not required Aqueous medium dissolves the phospholipids
Others	Ineffective process in removal of toxins and anti-nutritional factors	Highly efficient in removal of toxins and anti-nutritional factors
Limitations	Limitations are cited above	An additional de-emulsification step is required. High cost for enzyme production

**Fig. 2:**
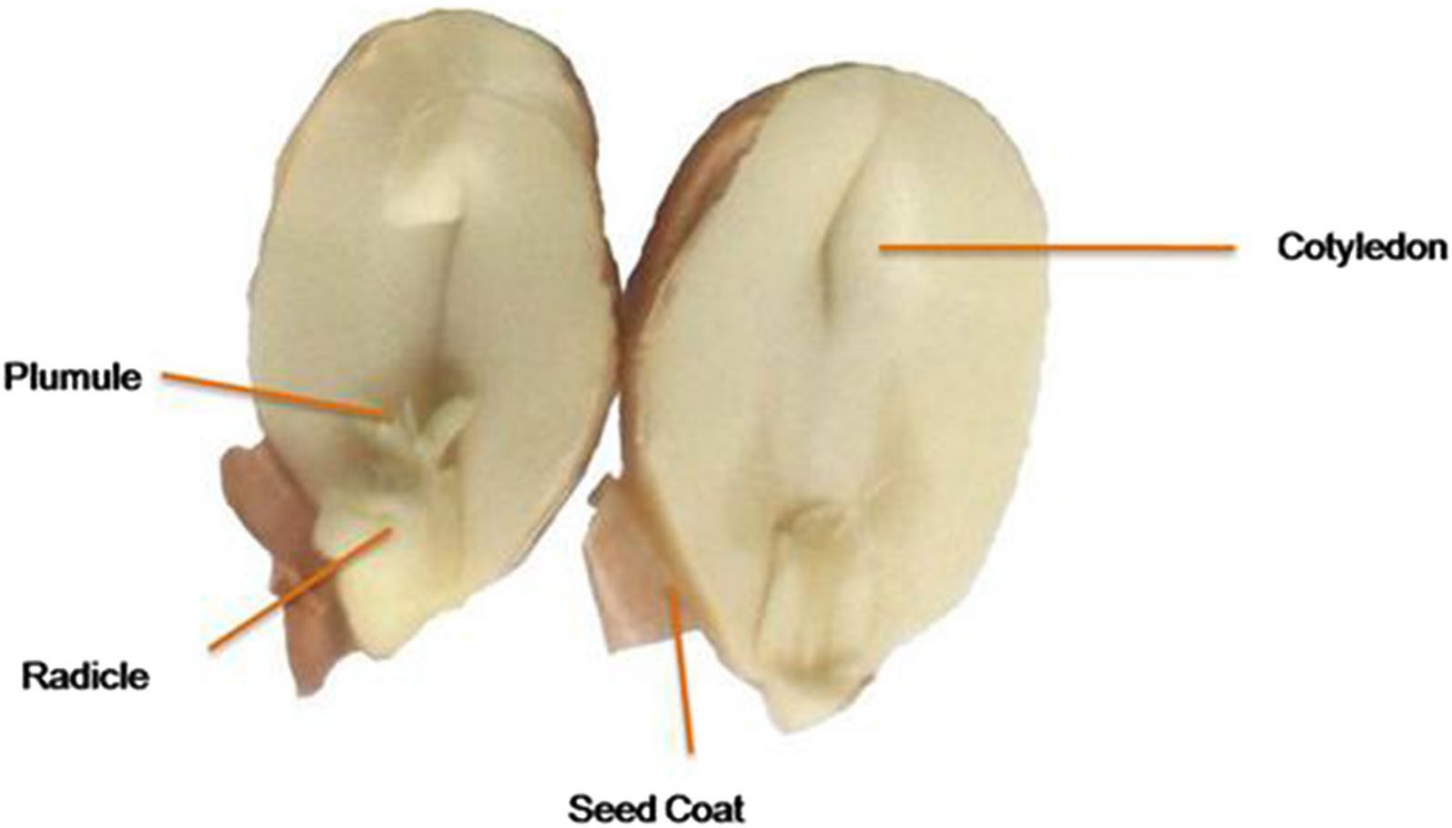
Diagram depicting the parts of groundnut oilseed

To know the role of enzymes on seed, the basic understanding of the architecture of crop oilseeds is indispensable. Oil seed cotyledon consists of discrete lipid and protein bodies which contains oil and protein respectively. In the cotyledons, proteins occupy a major proportion of 60–70% ranging in size from 2 to 20 µm in various oilseeds (Fig. 2) . Lipid bodies are the lipid reserves in fruits as well as in oilseeds. Their size varies from one species to another with an average range of 1–2 µm for most of the oilseeds. Microscopic structure of peanuts and soybean oilseeds depicts that the lipids are embedded with protein like cytoskeleton and the gaps are packed with lipids and cytoskeleton. These internal discrete cell organelles are surrounded by cell wall that is composed of cellulose, hemicelluloses, lignin and pectin.

### Selection of enzymes for oil extraction

Several factors are essential for the maximum recovery of oil from oilseeds. Application of enzymes either alone or in concoction can be determined based on the structure of oilseed, enzyme composition, type of enzyme, experimental conditions. For instance, heat treated soy bean flour separately treated with cellulase, pectinase, hemicellulase and protease (Alcalase 2.4 L from Bacillus licheniformis) enzymes, respectively. Among them, protease resulted higher yield (Alcalase 2.4 L) than rest of the enzymes [26]. Similarly, in extruded soybean flakes, protease treatment resulted higher yield of oil (96.0%) than phospholipase (73.4%) treatment [27]. Furthermore, when extruded soybean oil was treated with cellulase alone and with a mixture of cellulase and protease, no significant augmentation of soybean oil yields (68%) was observed. However, when the same oleaginous material was treated with protease it resulted in 88% of soybean oil [28]. It clearly elucidates that the hydrolysis of protein (which is in major proportion) in soybean by protease has succored the release of oil.

Similarly, rapeseed predominant with pectin in the cell wall was treated by pectinase that resulted 85.9% increase in oil yield [29]. On the other hand, some other research findings revealed that the application of enzyme mixtures have shown a better performance than individual enzymes presumably due to synergism [30]. For example, mixture of enzymes such as polygalacturonase, α-amylase and protease showed higher oil yield (80%) in coconut [31]. In contrary, soybean treated with combination of alcalase 2.4 L and viscozyme (a mixture of enzyme), no considerable increase in oil yield was observed [32]. The difference in activities of viscozyme can be attributed due to experimental conditions and the nature of oilseeds.

Consequently, these findings envisage for prior understanding of the architecture of targeted oilseed and selection of influential parameters to choose the best combination of enzymes. Hence, to achieve higher yields and recovery of co-products judicious use of enzymes is pre-requisite step. For optimization of the process, response surface methodology or genetic algorithm or any statistical methods could be employed to maximize the process by fixing the influential factors [14]. Several studies on application of enzymes either alone or in combination on different oilseeds for oil extraction have been presented in Table 2.

**Table 2:** Oil yield by enzymatic extraction method

Material	Enzymes applied	Oil yield (%)	References
Palm fruit	Pectinase/cellulase/tannase Tannase	35.90 12.70	[42]
Peanut (grounded)	Viscozyme L	13.10	[56]
Peanut (grounded)	Alcalase	42.86	[43]
	Protizyme™	24.43	[43]
Canola seeds (grounded)	Multifect CX 13L	09.50	[56]
Soybean flakes (extruded)	Multifect Neutral™	20.00	[50]
Rapeseed slurry	Pectinase	38.10	[28, 57]
Rapeseed slurry	Pectinase/cellulase/b-glucanase (4:1:1)	43.80	[28]
Soybean flakes (extruded)	Protease	13.40	[50]
*Moringa oleifera* seeds (grounded)	Neutrase 0.8 L/Termamyl	12.83	[49]

### Factors affecting enzyme mediated oil extraction

Aqueous enzymatic extraction (AEE) efficiency depends on several factors. In order to develop a viable process for oil extraction from oilseeds, factors responsible for the maximization have to be known to maintain the optimum conditions.

### Pre-treatment (grinding) of oleaginous materials

It is necessary to reduce the size of oleaginous materials (seeds/fruits) either by grinding or flaking to gain much access by enzymes. Grinding ruptures the cell constituents and releases the oil. In case of grinding, factors such as structural and chemical constituents of oilseed, initial moisture content are to be determined to make judicious choice either for wet or dry grinding [33]. Generally, oleaginous material with high moisture content can ground in wet condition, whereas for low moisture content oilseeds like rapeseed, peanut and soybean, drying is necessary. For example, grinding of coconut (high moisture content) in wet condition not only resulted higher oil yield but also alleviated drying step [34].

### Oilseeds particle size

Generally, lower particle size favors higher yield but scrawny seeds coupled with oleaginous material when treated with solvents may lose their microporosity that may result into unfavorable extraction due to non-uniform distribution. For instance, different particle sizes of linseed kernels improved the efficiency of oil extraction whereas with the same substrate showed inadequate oil recovery due to lack of enzymes access [30]. In addition, Rosenthe et al. [26] reported an increase of 31% yield when the particle size reduced from 400 to 100 µm [27].

### pH

Efficiency of oil extraction by enzyme depends mainly on pH factor. The extraction efficiency can be maximized at an optimum pH since each enzyme has a specific optimum value. Care should be taken to extract at far from the isoelectric point. Because, at specific isoelectric point of an enzyme, the protein is insoluble that might hamper the objective of oil extraction. For instance, a low yield of oil was observed in soybean, rapeseed, peanut and sunflower due to low solubility of protein at isoelectric point [35, 36]. To corroborate further, flaxseed oil yield was higher when treated with mixture of enzymes (cellulase, hemicellulase and pectinase, at a ratio of 1:1:1) at pH 4.5–5.0 than treatment with individual enzymes [32]. These studies envisage to maintain the pH at optimum level and to carry out the process far from the isoelectric point.

### Temperature

Temperature is another important factor for optimization of enzyme activity. Generally, enzyme activities are effective at or below 45 °C and increase in temperature results in denaturation of protein; as a result, it reduce the oil release from oilseeds [36]. Temperature has to be determined as per the quality of oilseed and fatty acids. For example, the congenial temperature for olive oil extraction is 30 °C and for linseed it is 34 °C, respectively. In a study conducted on peanut, the maximum yield was obtained at 40 °C, however, upon reduction of temperature to 37 °C resulted reduced yield [37]. Therefore, it is vital to optimize the temperature range as per the desired quality and the nature of the seed.

### Enzyme concentration/substrate ratio

Generally, the increase in concentration of enzyme leads to more interaction with substrate that consequently degrades the peptide bonds [38]. Increase in enzyme concentration until saturation of substrate active sites lead to more degradation of desired product and enhanced oil recovery. Additionally, increase beyond saturation levels may set off flavors, bitterness and carmelization of sugars which may hinder the oil extraction process [36, 39]. In addition, the cost of the enzyme (economics of the process) and quality of the oil are some other factors to consider before determination of the enzyme concentration [40].

### Oil:water ratio

Enzyme activity needs water or moisture content for several functions like diffusion, mobility of enzymes and hydrolytic reactions [41]. If an oleaginous material possesses low moisture content it leads to formation of thick suspension [29]. As a result, the enzyme action can be inhibited. On the other hand, if the oilseed contain higher moisture content it may dilute the enzyme and substrate concentrations which may feeble the reaction [42]. Hence, in order to have profound enzymatic reaction on the target, optimization of moisture content is inevitable.

### Shaking regime

Shaking or agitation regime helps in disruption of mechanical barriers (cell wall) and also perform uniform mixing of all contents in the reaction mixture [43]. Oil extraction from *Moringa oleifera* has been done at agitation speed of 50, 80 and 120 rpm, respectively. At 120 rpm, the oil droplets (bigger in size) were accumulated at the surface which has an advantage of easy separation [41]. In contrary, agitation is an energy driven process that may incur more cost on the process. In addition, it forms a stable emulsion that is cumbersome to separate [42].

### Challenges of green solvents and aqueous enzyme oil extraction (AEE)

Unprecedently, green solvents such as terpenes, IL’s and switchable solvents have huge potential to replace conventional solvent systems. Terpenes are gaining wide interest but scalability of the process is limited due to its high heat of vaporization, boiling point, density and viscosity. The problem could be avoided by extracting the solvents (terpenes) at low temperature and pressure using Clevenger apparatus. Generally, the bio-solvents are to be extracted by Clevenger apparatus at about 97–98 °C at atmospheric pressure. For instance, Sean et al. [44] have studied the quality of rice bran oil extraction with hexane and d-limonene solvents. The bio-solvent d-limonene is equivalent in terms of quality to that of hexane process [44]. Li et al. [21] has done similar studies of oil extraction from rapeseed. Hexane, ethanol, butanol, isopropanol, d-limonene, *p*-cymene and α-pinene were used to extract the oil from the rapeseed. Among the solvents, *p*-cymene obtained higher oil yield than the other solvents. The major oil components are free fatty acids (FFA), diglyceride (DAG), monoglyceride (MAG) and triglycerides, respectively. In *p*-cymene, the triglyceride content was low but high in free fatty acids, diglyceride and monoglyceride contents, respectively [21–44]. The result observed can be explained due to more polarity of the terpenes than hexane. Hence, it is intriguing that the terpenes can be a viable option to replace hexane and deploying this green solvent would ensure a cleaner environment, safer handling and non-toxicity.

Although aqueous enzyme oil extraction has huge potential, application of this technology is still hampered due to the factors such as high cost for enzyme production and downstream processing, long incubation time and unavoidable added step (de-emulsification) in the process. Nevertheless, due to the wide applications of AEE, commercial enzyme production has been expedited and as of now the enzyme production has become cheaper [38, 45]. Similarly, the downstream processing costs could be minimized by adapting suitable technologies than the conventional process. For instance, expanded bed affinity chromatography resulted 89% green fluorescent protein (GFP) with 2.7-purification fold using Ni^2+^ Streamline™, whereas Ni^2+^ alginate gave 91% of GFP recovery with 3.1-fold purification in a single step [46, 47]. Unlike chromatographic techniques, membrane technology has been employed to purify protein (penicillin acylase) from the cell lysate in a single step. Further, the specific enzyme activity has been confirmed by SDS-PAGE [48]. Moreover, several other techniques such as perfusion chromatography, affinity precipitation may be applied to make the process simpler with concomitant reduction in price [49, 50].

Another strategy for reducing the cost is enzyme immobilization, through which many cycles can be performed for oil extraction. The application of extracted cream emulsion, which possesses enzyme activity even after extraction, will certainly be a viable approach to reduce the cost. Cream emulsion is obtained in the process of AEE. Initially, the oleaginous material was pre-treated, extracted by solvent and separation leads to formation of oil and skim emulsion. It is reported that Protex 6L possessed 100% of activity in the fractions after extraction of oil [51]. Similarly, after extraction of oil from soybean around 84.7% of activity was observed in aqueous phases [52]. Apart from the above measures, AEE process saves energy by alleviating the necessity of solvent (used for stripping), process monitoring (in SE volatile compound emission has to be controlled) and simultaneous oil and protein recovery may compensate the challenges in the implementation of AEE [53–56].

## Conclusion

In the course of time, green solvents and technologies are in great demand because of environmental, health and energy issues. It is inevitable to develop a novel green technology for the oil extraction from various oilseeds. As each oilseed comprises of different architecture, the process needs to look for suitability of technology in economical and technical ways. In this review, green solvents coupled with AEE (green technology) not only ensure oil quality and protein extraction but also eco-friendly. In addition, they could reduce downstream processing steps. Furthermore, green solvents are effective in consumption of solvent, reduction of downstream processing steps (reclamation of solvent) without causing any effect to other desired products. AEE coupled with green solvents could be economical, eco-friendly and safer. Adoption of green technology and solvents is the need of an hour, as these are promising approaches for oil extraction towards environmental safety. However, further research findings should substantiate the viability of these approaches for the oil extraction from oilseeds.

## Abbreviations

AEE: aqueous enzyme oil extraction
DAG: diglyceride
FFA: free fatty acids
GS: green solvents
HSP: Hansen solubility properties
MAG: monoglyceride
